# Left Basal Ganglia Stroke-induced more Alterations of Functional Connectivity: Evidence from an fMRI Study

**DOI:** 10.2174/0115734056344477250222060225

**Published:** 2025-03-06

**Authors:** Qianqian Mao, Heng Wang, Jun Yao, Huiyou Chen, Yu-Chen Chen, Xindao Yin, Zhengqian Wang

**Affiliations:** 1 Department of Radiology, Nanjing First Hospital, Nanjing Medical University, Nanjing, China; 2 Department of Neurology, Nanjing Yuhua Hospital, Yuhua Branch of Nanjing First Hospital, Nanjing, China; 3 Department of Radiology, Lianshui County People’s Hospital, Huai’an, China

**Keywords:** Basal ganglia injury, Ischemic stroke, Resting-state functional magnetic resonance imaging, Dynamic functional network connectivity, Sliding window analysis, Dynamic functional connectivity

## Abstract

**Background:**

The basal ganglia area is a frequent site of stroke, which commonly causes intricate functional impairments. This study aims to uncover disparities in static and dynamic functional connectivity (FC) of the brain in patients afflicted with left-sided basal ganglia stroke (L-BGS) and right-sided basal ganglia region stroke (R-BGS), furthermore scrutinising the mechanism behind the lateralisation of the stroke.

**Methods:**

A total of 23 patients with L-BGS and 20 patients with R-BGS were recruited, alongside 20 healthy control subjects. Resting-state functional magnetic resonance imaging and sliding window techniques were employed to conduct static and dynamic FC analyses on both patient groups and controls, which can enable a more refined evaluation of the variations in neural signals.

**Results:**

The inter-network connectivity analysis showed significant changes only in the L-BGS patient group (*p <* 0.05). The R-BGS group showed increased connectivity in the auditory and posterior visual networks, while the L-BGS group showed reduced connectivity. In dynamic connectivity analyses, the L-BGS group exhibited greater positive network connectivity reorganization.

**Conclusion:**

Within one month of stroke onset, the L-BGS group showed a more pronounced impairment of inter-network connectivity, alongside enhanced FC compensatory changes of a positive nature. Differential changes in the two patient groups may provide useful information for individualized rehabilitation strategies.

## 1 INTRODUCTION

Ischaemic stroke is a notable worldwide health issue due to its high prevalence, disability and mortality rates, which cause a significant public health challenge that seriously impacts people in all countries [1]. Furthermore, it increases the socioeconomic burden [2]. To improve rehabilitation strategies for stroke patients, more attention has been given to enhancing the accuracy of functional outcome predictions after ischaemic stroke [3, 4]. The impact of infarction site on stroke patient functional outcomes is widely recognized [5]. The discrepancy in prognosis across different infarction sites may be attributed to lesion-induced injury and the subsequent disconnection of distant regions, which ultimately impacts network functionality [6, 7].

Consequently, prediction models should include consideration of the infarction site. The basal ganglia is a common stroke site and vascularly rich. It not only underlies complex movements but also regulates higher cognitive functions and non-motor complex behaviours that necessitate the processing and integration of diverse information sources [8]. Consequently, basal ganglia lesions frequently cause multifaceted functional impairments encompassing motor, attention, memory, visuospatial abilities, language, and executive functions [9]. In a previous multivariate analysis, it was demonstrated that among 901 patients presenting with small subcortical infarcts, the voxel-wise odds ratios for post-stroke cognitive impairment observed in the left thalamus was the highest, thereby indicating a high risk for the development of post-stroke cognitive impairment in the left thalamus [10]. This finding is closely related to the asymmetry of the human brain hemispheres. Cognitive functioning assessment following a stroke usually concentrates on language and memory, whereas the anatomical basis of verbal load, memory, and attention tends to be predominantly located in the left hemisphere. Consequently, the relationship between cognitive deficits and left hemisphere impairment is consistent with the expected brain-behaviour relationship. While the right basal ganglia region has not been identified as a primary source of cognitive impairment in earlier studies, it is crucial not to underestimate the risks of a right hemisphere stroke. Lateral spatial neglect and anosognosia are most commonly seen after right hemisphere stroke, both of which contribute to severe disability and may have negative effects on the recuperation of motor and cognitive function [11-13]. However, there has been limited research attention given to alterations in brain functional connectivity within the right basal ganglia region when exploring brain network studies in the basal ganglia region post-stroke. Furthermore, the discrepancies in brain functional connectivity within the basal ganglia region following stroke between the two hemispheres and the underlying neurobiological mechanisms remain uncertain.

Resting-state functional magnetic resonance imaging (fMRI) is a non-invasive technique for characterizing the intrinsic functional organization and connectivity patterns of the brain [14]. It serves as a valuable tool for investigating the mechanisms of vascular injury in stroke with disrupted blood oxygen signalling. This method can uncover abnormal mechanisms of other cortical regions and functional networks due to stroke [15]. However, conventional analytical tools assume that the brain maintains a “static” state of connectivity during the fMRI scanning period, typically lasting several minutes. This assumption makes it unfeasible to carry out a precise temporal assessment of fMRI signals, nor can we determine whether alterations in the functional networks' connectivity induced by stroke also exhibit short-term fluctuations [16]. To address this requirement, the use of 'dynamic' functional network connectivity (dFNC) analysis has arisen. This improves the temporal resolution of resting-state fMRI data, enabling the evaluation of connectivity changes within a few seconds [17, 18]. Dynamic measurements enable a more refined evaluation of the nature of abrupt variations in neural signals compared to static signals. Such variations are not only of behavioural significance [19, 20], but are also increasingly emerging as novel biomarkers of disease [21-23]. In a dynamic brain network study, changes in the temporal properties of large-scale network interactions were observed in post-stroke patients, and these changes were related to the severity of somatic deficits [24]. It is challenging to detect these findings using static analyses. Thus, dFNC analyses can evaluate the state of spontaneously formed connections in contrast to static functional connectivity analyses. Such analyses discern specific dynamic patterns that may be crucial in the neural reorganization process, determining the potential for brain recovery [25].

In this study, our focus is on the effects of left basal ganglia strokes (L-BGS) and right basal ganglia strokes (R-BGS) on other cortical regions and functional networks of the brain. Through comparisons, this investigation reveals differences in brain function for patients experiencing either left or right basal ganglia strokes. We hypothesise that there are static and dynamic functional connectivity differences in the neural networks of L-BGS and R-BGS patients and that the results in these static and dynamic functional connectivity analyses can provide new ideas and insights into the understanding and treatment of impaired brain function and neural circuits after unilateral basal ganglia strokes.

## 2 MATERIALS AND METHODS

### 2.1 Subjects and Clinical Data

This study retrospectively analyzed patients with first-ever acute unilateral stroke in the basal ganglia region (BGS) from July 2017 to September 2023. Participants ranged in age from 35 to 80 years and were all right-handed. All HCs were recruited from the local community. The National Institute of Health Stroke Scale (NIHSS), the Glasgow Coma Scale (GCS) and the Modified Rankin Scale (mRS) were used to assess the severity of stroke in each patient. Inclusion criteria were as follows: (1) age 35-80 years; (2) right-handed ; (3) examination within one month of stroke onset; (4) single lesion involvement; (5) NIHSS score <15 at onset; (6) conscious at onset with a GCS score of 15; (7) mRS score ≤3. Exclusion criteria were as follows: (1) those with contraindications to MRI (pacemakers, artificial heart valves, arterial clips, neurostimulators, other metal deposits in the body, and claustrophobia); (2) history of neurological or psychiatric disorders; (3) other cerebral abnormalities detected on the magnetic resonance images; (4) Displacement of head movement of more than 3 mm or rotation of more than 3° in MRI images. Two experienced imaging physicians diagnosed which side of the basal ganglia region the stroke occurred on by MRI images. Under the inclusion criteria, the study cohort comprised a total of 24 patients with L-BGS, 20 patients with R-BGS, and 20 HCs. During the post-processing phase, one patient with L-BGS was excluded due to the presence of head movement artefacts on the functional magnetic resonance images. Finally, 23 patients with L-BGS and 20 HCs were included in the left lesion analysis and 20 patients with R-BGS and 20 HCs were included in the right lesion analysis. The study adhered to the Declaration of Helsinki, was approved by the Ethics Committee of Nanjing First Hospital, stroke patients were exempted from informed consent, and all HCs signed an informed consent form.

### 2.2 MRI Acquisition

A 3.0T Philips magnetic resonance scanner (Ingenia, Philips Medical Systems, The Netherlands) and an 8-channel receiver array magnetic head coil were used to acquire post-stroke MRI data. Foam padding and earplugs were used to minimize head movement and scanner noise. Subjects were instructed to remain still during image acquisition, not to fall asleep, and to avoid any head movement during scanning. Functional imaging was performed using a gradient-echo planar imaging sequence: repetition time TR = 2000 ms; echo time TE = 30 ms; number of slices = 36; thickness = 4 mm; gap = 0 mm; field of view = 240 mm × 240 mm; acquisition matrix = 64 × 64; flip angle = 90°. The functional MRI sequence took 8 minutes and 8 seconds. The structural image was acquired using a high-resolution 3D fast echo T1WI sequence with repetition/echo time=8.2/3.8 ms, number of slices=170, thickness=1 mm, gap=0 mm, flip angle=8°, acquisition matrix=256×256, and field of view=256 mm×256 mm. The acquisition time was 5 minutes and 29 seconds.

### 2.3 MRI Data Pre-processing

Preprocessing of fMRI data was performed using the data processing toolbox Gretna (GRETNA 2.0; https://www.nitr c.org/projects/gretna/) based on the SPM12 software package (https://www.fil.ion.ucl.ac.uk/spm/) on the Matlab R2013b platform (Matlab R2013b; https://www.mathworks.com/ products/matlab.html). Specific steps of data preprocessing for static functional connectivity analyses included (A) removing the first 10 volumes from each time series to account for the time required for participants to adapt to the scanning environment. (B) Temporal layer correction allowed different layers of data to be acquired at the same time. (C) Head motion correction for realignment. Data from subjects showing head motion >3.0 mm translation or >3.0° rotation were excluded from the analysis. One patient with L-BGS was excluded because of a translational head movement >3 mm. (D) The remaining dataset was spatially normalized to the Montreal Neurological Institute template (resampled voxel size = 3 × 3 × 3 mm^3^). (E) Spatial smoothing was performed using an 8 mm Gaussian kernel. Dynamic functional connectivity analyses, on the other hand, performed preprocessing steps (A) to (D), omitting step (E) smoothing, in addition to denormalization trending and band-pass filtering (0.01-0.08 Hz) to reduce the effects of low-frequency drift and high-frequency noise. Subsequently, signals including head movements as well as those from cerebrospinal fluid and white matter were regressed from the data.

### 2.4 Static Functional Network Connectivity Analysis

#### 2.4.1 Independent Component Analysis

Independent Component Analysis (ICA) was performed using Group ICA of the FMRI Toolbox software (Medical Image Analysis Lab, University of New Mexico, Albuquerque, NM, USA; http://icatb.sourceforge.net/). ICA was performed in three steps:(A) Data reduction. (B) Application of the Infomax ICA algorithm with stability analysis using the REGULAR method. (C) Inverse reconstruction for each subject using the GICA algorithm. The number of components for the validation analyses varied from 50 to 40 and then to 30, and data simplification was followed by a series of spatial ICAs performed on the aggregated data of the participants, which finally yielded our optimal number of independent component (IC) evaluations of 24. To obtain robust and accurate results, the number of independent components was set at 24, and the number of group ICA runs in the fMRI toolbox was chosen to be 20. Each independent connection strength value within a component was converted into a Z-score to reflect the degree of correlation between the time series of a given voxel and the average time series of its corresponding component.

We refer to the section on the inverse reconstruction algorithm for group-level independent component analysis in a previous study, using 11 network components from a healthy sample [26, 27], the 11 network components were (1) auditory network (AN), (2) dorsal attention network(DAN), (3) left frontoparietal network (lFPN), (4) right frontoparietal network (rFPN), (5) occipital pole visual network (pVN), (6) posterior default mode network (pDMN), (7) dorsal sensorimotor network (dSMN), (8) lateral visual network (lVN), (9) medial visual network (mVN), (10) anterior default mode network (aDMN), and (11) ventral sensorimotor network (vSMN).

#### 2.4.2 Analysis of Intra-network Connectivity

One-sample *t*-test were performed on the 6 network components using SPM12 (https://www.fil.ion.ucl.ac.uk/spm/), saving only significant voxels, which were used to obtain masks for subsequent analyses. The threshold was set at *p <* 0.001 and corrected by FDR. Two groups of patients and HCs were compared separately using a two-sample *t*-test (left basal ganglia stroke-healthy control, right basal ganglia stroke-healthy control), with age and sex included as covariates to avoid the potential effect of these confounders. The statistical threshold for FDR correction for multiple comparisons was set at *p <* 0.001.

#### 2.4.3 Analysis of Inter-network Connectivity

To assess static connectivity, pairwise Pearson correlations were calculated for each participant's Z-transformed time course, with 15 pairs of connectivity values per participant. Patients with L-BGS and patients with R-BGS were compared separately with HCs, and we assessed differences in static functional network connectivity using two-sample t-test analyses (L-BGS-HCs, R-BGS-HCs; significant level *p* < 0.05, corrected for FDR).

### 2.5 Analysis of Dynamic Functional Network Connectivity

The sliding window technique was employed using the GIFT Toolbox (Group ICA of fMRI Toolbox, http://trendscenter.org/software/gift) to produce the dynamic connectivity matrix of each participant. This method was utilized to facilitate the exploration and characterization of the dynamic qualities of the brain's functional connectivity. The k-means clustering method was employed to derive the spontaneous states of the brain and analyse their properties in a statistically sound manner. This was accomplished by defining 191 individual time windows, each lasting for 30 repetition times (TR), through the use of a sliding time window. The window length of 30 TR was chosen based on previous studies confirming that a window length of 20-30 TR better reflects the dynamic characteristics of the brain [28, 29]. These time windows were convolved with Gaussian functions and stepped by 1 TR. In each of these windows, the dynamic connectivity matrix was computed using the sparse inverse covariance matrix. The functional connectivity matrix underwent Fisher's Z-transform to acquire Z-values for stabilizing the variance for in-depth analysis.

The k-mean cluster approach was utilized to achieve spontaneous states of the subject group. A median matrix, preserving the dynamic connectivity matrix of each patient group, was extracted based on the state for the consecutive stage of contiguous edge investigation. Comparisons between L-BGS-HCs, R-BGS-HCs were performed separately along each state using a two-sample *t*-test (*p* < 0.05, FDR corrected).

### 2.6 Statistical Analyses

Differences in demographic information and clinical measures among the three groups were analyzed in one-sided analyses using one-way analysis of variance (ANOVA) followed by post-hoc tests (t-test for means and chi-square test), and non-parametric tests were used for gender, NIHSS, GCS scores and volume of lesions for L-BGS and R-BGS. Results are reported at the *p* < 0.05 level of significance. Statistical analyses were performed using SPSS 26.0 (SPSS, Chicago, IL, USA). For intra-network functional connectivity outcome analyses, we used a two-sample *t*-test (L-BGS-HCs, R-BGS-HCs; *p* < 0.001, clump level FDR correction). For the analysis of inter-network functional connectivity results, we assessed static functional network connectivity differences with two-sample *t*-test analyses (L-BGS-HCs, R-BGS-HCs; significant level *p* < 0.05, FDR-corrected). For cluster analysis results of dynamic network functional connectivity, we performed two-sample *t*-test (L-BGS-HCs, R-BGS-HCs; significant level *p* < 0.05, corrected for FDR). Age and gender were regressed as covariates in the above statistical analyses.

## 3 RESULTS

### 3.1 Subjects and Clinical Data

The characteristics of L-BGS patients, R-BGS and HCs are summarised in Table **1**. A total of 23 patients with L-BGS, 20 patients with R-BGS, and 20 HCs were included in this study. ANOVA analysis and chi-squared test followed by two two comparisons showed that there was no statistically significant difference between the two patient groups in terms of age, gender, NHISS scores, GCS scores volume of lesions. There was no statistically significant difference between the age and gender ratios of the two patient groups and HCs (*p* > 0.05). Age and sex were regressed as covariates in all analyses.

### 3.2 Static Functional Network Connectivity Analysis

Following the reverse reconstruction of ICA, 5 of the 11 independent components were excluded due to spatial inaccuracies and low ratios of low to high-frequency spectra. The remaining 6 network components were (1) AN, (2) DAN, (3) lFPN, (4) rFPN, (5) pVN, and (6) pDMN.

#### 3.2.1 Analysis of Intra-network Connectivity

In intra-network functional connectivity analysis, we analysed the differences between the two groups of patients based on the signal intensity of each voxel within the six spatial maps of brain networks screened by the previous independent component analysis, providing further evidence of changes in network connectivity in the basal ganglia region in the early post-stroke period. Compared to HCs, L-BGS patients had increased connectivity within the three brain networks DAN, pDMN and rFPN and decreased connectivity within the two networks DAN and rFPN (*p* < 0.001, corrected for cluster-level FDR) (Tables **2** and **3**), whereas R-BGS patients had increased connectivity within the four brain networks AN, DAN, pDMN and pVN compared to HCs (*p* < 0.001, corrected for cluster-level FDR) (Table **4**).

#### 3.2.2 Analysis of Inter-network Connectivity

**Fig. (1):**
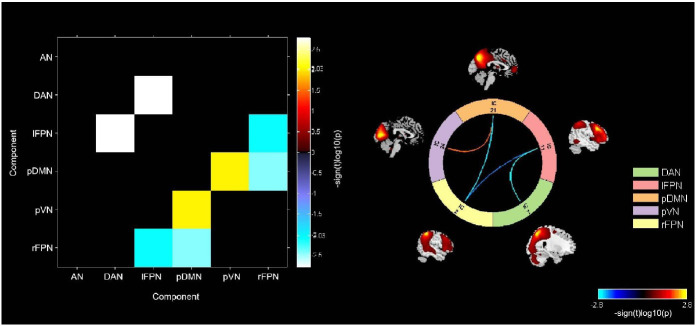
Significant differences in inter-network functional connectivity between L-BGS and HCs. Compared with HCs, L-BGS patients had reduced strength of connectivity between the lFPN and the pDMN, rFPN, and between the lFPN and the DAN; and enhanced connectivity between the pVN and the pDMN (*p* < 0.05, corrected for FDR). pDMN: posterior default mode network; lFPN: left frontoparietal network; rFPN: right frontoparietal network; pVN: occipital pole visual network; DAN: dorsal attention network; L-BGS: left-sided basal ganglia stroke; HC: healthy control.

The difference between L-BGS patients and HCs was statistically significant, with L-BGS patients having predominantly reduced connectivity between networks, as well as enhanced connectivity in individual brain regions. Compared with HCs, L-BGS patients had reduced connectivity strength between lFPN and pDMN, lFPN and rFPN, and between lFPN and DAN; and enhanced connectivity strength between pVN and pDMN (*p* < 0.05, corrected for FDR) (Fig. **1**). When statistically comparing R-BGS patients with HCs, we did not obtain significant differences (*p* < 0.05, corrected for FDR).

### 3.3 Analysis of Dynamic Functional Network Connectivity

**Fig. (2):**
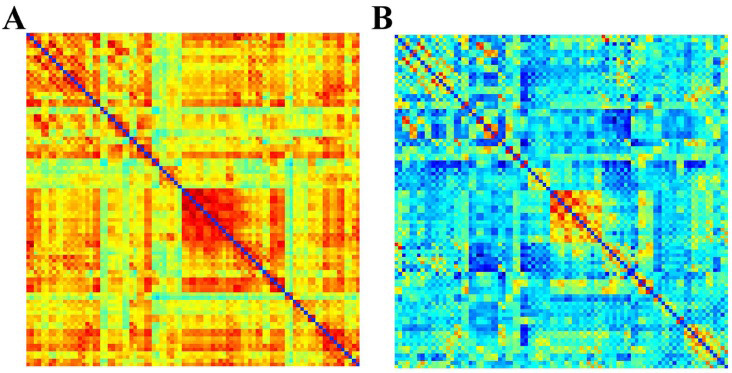
Two states of connectivity. (**A**) Total frequency of state 1: 41.29%. State 1 has highly positive connectivity, and we call this state the strongly connected state; (**B**) Total frequency of state 2: 58.71%. State 2 is dominated by negative connectivity and we call this state a weakly connected state.

Next, we examined dynamic functional network connectivity (dFNC). By applying the k-mean clustering algorithm to an estimated 191 functional connectivity matrices per subject and the optimization criteria for the number of states described above, we identified two connectivity states that recurred across subjects during functional MRI scans. The states were represented and described in the order given by k-mean clustering. The first connectivity state has highly positive connectivity. The first connectivity state has highly positive connectivity. We refer to this state as the strongly connected state (total frequency of state 1: 41.29%). The second connectivity state has predominantly negative connectivity, which we call the weakly connected state (total frequency of state 2: 58.71%) (Fig. **2**).

**Fig. (3):**
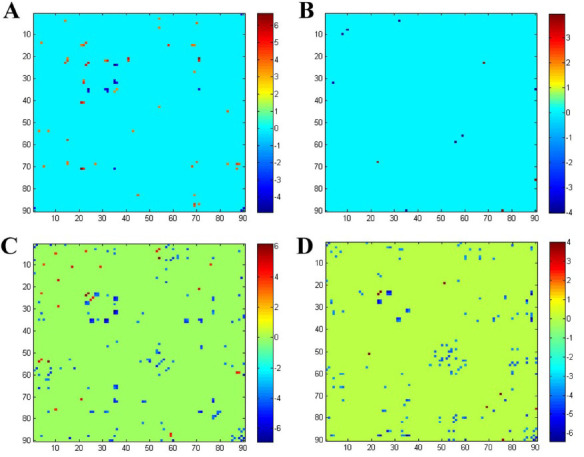
Significant differences in functional network connectivity between the two groups of patients with impaired basal ganglia regions compared to HCs in two states, respectively. (**A**) In state 1, L-BGS patients showed significant connectivity edges dominated by enhanced connectivity; (**B**) In state 1, R-BGS patients showed significantly fewer significant connectivity edges and predominantly weakened connectivity; (**C**) In state 2, L-BGS patients showed significant connectivity edges dominated by weakened connectivity; and (**D**) in state 2, patients with R-BGS show significant edges mainly in the form of weakened connectivity. L-BGS: left-sided basal ganglia stroke; R-BGS: right-sided basal ganglia stroke; HC: healthy control.

In the next connectivity analysis, significant differences were found in the two-sample *t*-test analysis (*p* < 0.05, FDR-corrected), where, compared to HCs, in the strongly connected state, L-BGS patients showed significant connectivity edges dominated by enhanced connectivity, whereas the R-BGS showed significantly fewer significant connectivity edges and predominantly weakened connectivity; and in the weakly connected state, both groups showed significant connectivity edges dominated by weakened connectivity (Fig. **3**).

## 4 DISCUSSION

When dealing with resting-state MRI data, the use of dynamic functional connectivity analysis methods leads to more fine-grained conclusions in the time domain [29, 30]. In this study, we analyzed the dynamic and static connectivity behavior of patients with L-BGS and patients with R-BGS within one month after acute stroke onset. We considered the effect of the degree of damage to the left and right basal ganglia regions on connectivity, and there was no significant difference in the degree of damage between the two groups based on the areas of damage reflected on imaging (both restricted to the basal ganglia region) and assessed by the NIHSS. Despite similar levels of clinical damage in both groups, patients with damage to the basal ganglia region in the left and right groups showed significant differences compared with healthy controls.

### 4.1 Differences in Static Brain Network Functional Connectivity

In the static brain network connectivity analysis, only patients with L-BGS showed altered inter-network connectivity mainly characterised by reduced inter-network connectivity, which is associated with cognitive function in previous studies [31, 32], and our findings may suggest that L-BGS patients showed more altered brain inter-network connectivity early in the stroke onset, which is consistent with previous studies mentioning that L-BGS is strongly associated with cognitive impairment (10). Relatively, patients with L-BGS also showed increased connectivity between brain networks, which seems to be a compensatory change [33].

In contrast, in the analysis of functional connectivity within static brain networks, increased connectivity within the AN and pVN was found only within the R-BGS patient group, whereas diminished connectivity within the DAN and rFPN was found only within the L-BGS patient group. This seems to suggest that L-BGS patients have a more extensive impairment of intra-network connectivity compared to R-BGS patients. In addition, this difference seems to suggest that different brain networks and brain functions are affected when the lesions occur on different sides of the basal ganglia region, and this lateralized alteration may contribute to the different clinical symptoms. Previous studies have found that stroke in the basal ganglia region causes impairments in cognitive function, motor function, executive function and vision [34-36]. These disorders can lead to complex changes in brain function involving multiple brain regions [37], resulting in the reorganization of functional networks [38, 39]. The functional connectivity changes we observed within the pVN, AN, pDMN and frontoparietal network (FPN) may be closely related to these impairments. In particular, changes in the FPN have previously been found to cause changes in FC within the FPN after stroke in the basal ganglia region, which seem to indicate changes in cognitive function [8, 32-34, 40, 41], but reduced functional connectivity within the FPN was only observed in the group of patients with L-BGS, which may suggest that cognitive changes are more pronounced in patients with L-BGS compared to those with R-BGS. It should be noted that the region of increased FC within the FPN in the L-BGS group was mainly located in the contralateral hemisphere, which can be considered as a compensatory mechanism to offset the damage caused by the damaged brain region [42].

Previous studies have shown that the most common symptoms after right hemisphere stroke are lateral spatial neglect and anosognosia [11-13]. The increased connectivity within the pVN in the group of R-BGS patients identified in our present study suggests that the brain has already experienced altered functional connectivity within the visual network (VN) within one month after the onset of R-BGS. This may provide evidence for an intrinsic mechanism for the high prevalence of lateralized spatial neglect after right hemisphere stroke.

### 4.2 Differences in Dynamic Brain Networks' Functional Connectivity

In the dynamic connectivity analysis, there were also significant differences in the performance of the connectivity state between the two patient groups in different connectivity states. In the weak connectivity state, both groups showed significant connectivity edges dominated by weak connectivity, and the number of significant connectivity edges was significantly higher than in the strong connectivity state, which may reflect the manifestation of functional brain injury in patients after stroke, which is still dominated by weak connectivity in the early stage of stroke. In the strong connectivity state, on the other hand, we found significantly more changes in connectivity edges in the L-BGS group than in the R-BGS group, and this was mainly manifested by connectivity enhancement. This high degree of connectivity enhancement is reminiscent of the hyperactivation and over-recruitment of cortical regions found in previous studies [43], and importantly, previous researchers have noted that these highly connectivity-enhanced changes represent early signs of reorganization [44].

The dynamic pattern observed here therefore seems to suggest that these increases in functional connectivity in the cortex are aimed at restoring lost brain function [45]. It is also suggested that patients with L-BGS show a more active reorganization of network connectivity compared to patients with R-BGS. These two specific patterns were not identified and described in the previous static analyses, as they were only inferred in our dynamic analyses. The different findings of static FC (sFC) and dynamic FC (dFC) may stem from the essential differences in their analytical approaches. sFC focuses on the average strength of connections between brain regions in the resting state, whereas dFC focuses on analysing changes in the strength of these connections over time. Thus, sFC may reflect more the static structural relationships between brain regions, whereas dFC is more revealing of the dynamic nature of brain functional activity. In the future, these findings may have a role in guiding clinical treatments when using non-invasive brain stimulation as a therapeutic modality to induce cortical plasticity to promote functional recovery [46, 47].

### 4.3 STUDY LIMITATIONS

(1) the sample size of this study is small, which may lead to biased results, and further expansion of the sample size is needed. (2) this study only included the analysis of NIHSS scores and did not include a comprehensive neuropsy-chological assessment to evaluate the differences in cognitive aspects such as orientation, executive function, visuospatial, phonology, memory, etc. between the left and right basal ganglia region after stroke, and we will further analyse these indexes in the future to see whether there is also a bias in basal ganglia function after stroke. (3) We mainly investigated the biased lateralization of brain network connectivity in patients within one month after the onset of stroke in the basal ganglia region, and how this difference changes with the recovery of stroke patients is also a direction of interest for our future research.

## CONCLUSION

In conclusion, the present study confirms that there are significant differences in brain network functional connectivity changes between L-BGS patients and R-BGS patients within 1 month of stroke onset. L-BGS patients showed more pronounced impairment in brain network connectivity and more positive compensatory changes in brain network connectivity reorganization and connectivity enhancement. Changes in functional brain network connectivity in early stroke patients with lateralization may provide a neurobiological mechanism to explain clinical symptoms. In addition, the use of dynamic connectivity methods provided further insight into changes in functional brain connectivity after stroke, revealing changes in the reorganization of brain network connectivity in different connectivity states when lesions are located in different hemispheres.

## Figures and Tables

**Table 1:** Clinical data of the case and control groups.

**Items**	**L-BGS(n=23)**	**R-BGS(n=20)**	**HC(n=20)**	***p***
Age, years	57.70±9.77	63.65±7.64	59.75±6.76	0.156
Gender: male/female	16/7	16/4	9/11	0.058
NIHSS score	2.30±1.79	2.95±2.28	-	0.374
GCS score	15	15	-	1
Volume of lesions, mm^3^	1031.67±241.18	1116.67±285.27		0.981

**Note:**Data are presented as ±standard deviation. **Abbreviations:** L-BGS: left-sided basal ganglia stroke; R-BGS: right-sided basal ganglia stroke; HC, healthy control; NIHSS: National Institute of Health Stroke Scale; GCS: Glasgow Coma Scale

**Table 2:** Brain regions with significantly increased FC values in the L-BGS group compared with HC.

**RNS**	**Brain Region**	**Peak Location MNI (mm)**			**T-score**	**Cluster Size (voxels)**
		**x**	**y**	**z**		
DAN	Cuneus_L	-3	-81	21	5.64	76
	Occipital_Sup_R	27	-81	27	6.04	300
	Angular_L	-36	-69	39	6.31	44
	Precuneus_R	-27	-39	57	7.23	379
pDMN	Cingulum_Mid_L	-3	6	36	5.02	44
rFPN	Calcarine_R	15	-72	3	5.46	127

**Abbreviations**RNS: resting-state network; MNI: Montreal Neurological Institute; L-BGS: left-sided basal ganglia stroke; HC: healthy control; DAN: dorsal attention network; pDMN: posterior default mode network; rFPN: right frontoparietal network;Cuneus_L: Cuneate lobe; Occipital_Sup_R: Supraoccipital gyrus; Angular_L: angular gyrus; Precuneus_R: Anterior cuneate lobe; Cingulum_Mid_L: Medial and paracingulate gyrus; Calcarine_R: Perichondrial cortex.

**Table 3:** Brain regions with significantly lower FC values in the L-BGS group compared with HC.

**RNS**	**Brain Region**	**Peak Location MNI (mm)**			**T-score**	**Cluster Size (voxels)**
		**x**	**y**	**z**		
DAN	Frontal_Inf_Oper_R	48	12	30	-4.63	62
rFPN	Parietal_Inf_L	-51	-45	45	-5.06	154
	Parietal_Inf_R	57	-42	42	-5.59	201

**Abbreviations:**RNS: resting-state network; MNI: Montreal Neurological Institute; L-BGS: left-sided basal ganglia stroke; HC: healthy control; DAN: dorsal attention network; rFPN: right frontoparietal network; Frontal_Inf_Oper_R: Insular inferior frontal gyrus; Parietal_Inf_L: Inferior parietal angular gyrus; Parietal_Inf_R: Inferior parietal angular gyrus.

**Table 4:** Brain regions with significantly increased FC values in the R-BGS group compared with HC.

**RNS**	**Brain Region**	**Peak Location MNI (mm)**			**T-score**	**Cluster Size (voxels)**
		**x**	**y**	**z**		
AN	Temporal_Sup_L	-45	21	3	4.82	107
DAN	Occipital_Sup_R	24	-78	33	7.33	152
	Precuneus_R	15	-42	48	6.04	76
	Postcentral_L	-24	-45	51	6.59	70
pDMN	Fusiform_L	-27	-33	-21	4.85	53
	Occipital_Mid_R	27	-72	30	5.88	93
pVN	Calcarine_R	24	-75	9	6.65	465

**Abbreviations:** RNS: resting-state network; MNI: Montreal Neurological Institute; R-BGS: right-sided basal ganglia stroke; HC: healthy control; AN: auditory network; DAN: dorsal attention network; pDMN: posterior default mode network; pVN: occipital pole visual network(pVN); Temporal_Sup_L: Superior temporal gyrus; Occipital_Sup_R: Supraoccipital gyrus; Precuneus_R: Anterior cuneate lobe; Postcentral_L:Posterior central gyrus; Fusiform_L: Fusiform gyrus; Occipital_Mid_R: Mid-occipital gyrus; Calcarine_R: Perichondrial cortex.

## Data Availability

The data and supportive information is available within the article.

## LIST OF ABBREVIATIONS

L-BGS: Left-sided basal ganglia stroke
R-BGS: Right-sided basal ganglia region stroke
dFNC: 'dynamic' functional network connectivity
fMRI: Functional magnetic resonance imaging
ICA: Independent Component Analysis

## STANDARDS OF REPORTING

STROBE guidelines were followed.

## References

[1] Feigin V.L., Stark B.A., Johnson C.O., Roth G.A., Bisignano C., Abady G.G., Abbasifard M., Abbasi-Kangevari M., Abd-Allah F., Abedi V., Abualhasan A., Abu-Rmeileh N.M.E., Abushouk A.I., Adebayo O.M., Agarwal G., Agasthi P., Ahinkorah B.O., Ahmad S., Ahmadi S., Ahmed Salih Y., Aji B., Akbarpour S., Akinyemi R.O., Al Hamad H., Alahdab F., Alif S.M., Alipour V., Aljunid S.M., Almustanyir S., Al-Raddadi R.M., Al-Shahi Salman R., Alvis-Guzman N., Ancuceanu R., Anderlini D., Anderson J.A., Ansar A., Antonazzo I.C., Arabloo J., Ärnlöv J., Artanti K.D., Aryan Z., Asgari S., Ashraf T., Athar M., Atreya A., Ausloos M., Baig A.A., Baltatu O.C., Banach M., Barboza M.A., Barker-Collo S.L., Bärnighausen T.W., Barone M.T.U., Basu S., Bazmandegan G., Beghi E., Beheshti M., Béjot Y., Bell A.W., Bennett D.A., Bensenor I.M., Bezabhe W.M., Bezabih Y.M., Bhagavathula A.S., Bhardwaj P., Bhattacharyya K., Bijani A., Bikbov B., Birhanu M.M., Boloor A., Bonny A., Brauer M., Brenner H., Bryazka D., Butt Z.A., Caetano dos Santos F.L., Campos-Nonato I.R., Cantu-Brito C., Carrero J.J., Castañeda-Orjuela C.A., Catapano A.L., Chakraborty P.A., Charan J., Choudhari S.G., Chowdhury E.K., Chu D-T., Chung S-C., Colozza D., Costa V.M., Costanzo S., Criqui M.H., Dadras O., Dagnew B., Dai X., Dalal K., Damasceno A.A.M., D’Amico E., Dandona L., Dandona R., Darega Gela J., Davletov K., De la Cruz-Góngora V., Desai R., Dhamnetiya D., Dharmaratne S.D., Dhimal M.L., Dhimal M., Diaz D., Dichgans M., Dokova K., Doshi R., Douiri A., Duncan B.B., Eftekharzadeh S., Ekholuenetale M., El Nahas N., Elgendy I.Y., Elhadi M., El-Jaafary S.I., Endres M., Endries A.Y., Erku D.A., Faraon E.J.A., Farooque U., Farzadfar F., Feroze A.H., Filip I., Fischer F., Flood D., Gad M.M., Gaidhane S., Ghanei Gheshlagh R., Ghashghaee A., Ghith N., Ghozali G., Ghozy S., Gialluisi A., Giampaoli S., Gilani S.A., Gill P.S., Gnedovskaya E.V., Golechha M., Goulart A.C., Guo Y., Gupta R., Gupta V.B., Gupta V.K., Gyanwali P., Hafezi-Nejad N., Hamidi S., Hanif A., Hankey G.J., Hargono A., Hashi A., Hassan T.S., Hassen H.Y., Havmoeller R.J., Hay S.I., Hayat K., Hegazy M.I., Herteliu C., Holla R., Hostiuc S., Househ M., Huang J., Humayun A., Hwang B-F., Iacoviello L., Iavicoli I., Ibitoye S.E., Ilesanmi O.S., Ilic I.M., Ilic M.D., Iqbal U., Irvani S.S.N., Islam S.M.S., Ismail N.E., Iso H., Isola G., Iwagami M., Jacob L., Jain V., Jang S-I., Jayapal S.K., Jayaram S., Jayawardena R., Jeemon P., Jha R.P., Johnson W.D., Jonas J.B., Joseph N., Jozwiak J.J., Jürisson M., Kalani R., Kalhor R., Kalkonde Y., Kamath A., Kamiab Z., Kanchan T., Kandel H., Karch A., Katoto P.D.M.C., Kayode G.A., Keshavarz P., Khader Y.S., Khan E.A., Khan I.A., Khan M., Khan M.A.B., Khatib M.N., Khubchandani J., Kim G.R., Kim M.S., Kim Y.J., Kisa A., Kisa S., Kivimäki M., Kolte D., Koolivand A., Koulmane Laxminarayana S.L., Koyanagi A., Krishan K., Krishnamoorthy V., Krishnamurthi R.V., Kumar G.A., Kusuma D., La Vecchia C., Lacey B., Lak H.M., Lallukka T., Lasrado S., Lavados P.M., Leonardi M., Li B., Li S., Lin H., Lin R-T., Liu X., Lo W.D., Lorkowski S., Lucchetti G., Lutzky Saute R., Magdy Abd El Razek H., Magnani F.G., Mahajan P.B., Majeed A., Makki A., Malekzadeh R., Malik A.A., Manafi N., Mansournia M.A., Mantovani L.G., Martini S., Mazzaglia G., Mehndiratta M.M., Menezes R.G., Meretoja A., Mersha A.G., Miao Jonasson J., Miazgowski B., Miazgowski T., Michalek I.M., Mirrakhimov E.M., Mohammad Y., Mohammadian-Hafshejani A., Mohammed S., Mokdad A.H., Mokhayeri Y., Molokhia M., Moni M.A., Montasir A.A., Moradzadeh R., Morawska L., Morze J., Muruet W., Musa K.I., Nagarajan A.J., Naghavi M., Narasimha Swamy S., Nascimento B.R., Negoi R.I., Neupane Kandel S., Nguyen T.H., Norrving B., Noubiap J.J., Nwatah V.E., Oancea B., Odukoya O.O., Olagunju A.T., Orru H., Owolabi M.O., Padubidri J.R., Pana A., Parekh T., Park E-C., Pashazadeh Kan F., Pathak M., Peres M.F.P., Perianayagam A., Pham T-M., Piradov M.A., Podder V., Polinder S., Postma M.J., Pourshams A., Radfar A., Rafiei A., Raggi A., Rahim F., Rahimi-Movaghar V., Rahman M., Rahman M.A., Rahmani A.M., Rajai N., Ranasinghe P., Rao C.R., Rao S.J., Rathi P., Rawaf D.L., Rawaf S., Reitsma M.B., Renjith V., Renzaho A.M.N., Rezapour A., Rodriguez J.A.B., Roever L., Romoli M., Rynkiewicz A., Sacco S., Sadeghi M., Saeedi Moghaddam S., Sahebkar A., Saif-Ur-Rahman K.M., Salah R., Samaei M., Samy A.M., Santos I.S., Santric-Milicevic M.M., Sarrafzadegan N., Sathian B., Sattin D., Schiavolin S., Schlaich M.P., Schmidt M.I., Schutte A.E., Sepanlou S.G., Seylani A., Sha F., Shahabi S., Shaikh M.A., Shannawaz M., Shawon M.S.R., Sheikh A., Sheikhbahaei S., Shibuya K., Siabani S., Silva D.A.S., Singh J.A., Singh J.K., Skryabin V.Y., Skryabina A.A., Sobaih B.H., Stortecky S., Stranges S., Tadesse E.G., Tarigan I.U., Temsah M-H., Teuschl Y., Thrift A.G., Tonelli M., Tovani-Palone M.R., Tran B.X., Tripathi M., Tsegaye G.W., Ullah A., Unim B., Unnikrishnan B., Vakilian A., Valadan Tahbaz S., Vasankari T.J., Venketasubramanian N., Vervoort D., Vo B., Volovici V., Vosoughi K., Vu G.T., Vu L.G., Wafa H.A., Waheed Y., Wang Y., Wijeratne T., Winkler A.S., Wolfe C.D.A., Woodward M., Wu J.H., Wulf Hanson S., Xu X., Yadav L., Yadollahpour A., Yahyazadeh Jabbari S.H., Yamagishi K., Yatsuya H., Yonemoto N., Yu C., Yunusa I., Zaman M.S., Zaman S.B., Zamanian M., Zand R., Zandifar A., Zastrozhin M.S., Zastrozhina A., Zhang Y., Zhang Z-J., Zhong C., Zuniga Y.M.H., Murray C.J.L. (2021). Global, regional, and national burden of stroke and its risk factors, 1990–2019: A systematic analysis for the Global Burden of Disease Study 2019.. Lancet Neurol..

[2] Gerstl J.V.E., Blitz S.E., Qu Q.R., Yearley A.G., Lassarén P., Lindberg R., Gupta S., Kappel A.D., Vicenty-Padilla J.C., Gaude E., Atchaneeyasakul K.C., Desai S.M., Yavagal D.R., Peruzzotti-Jametti L., Patel N.J., Aziz-Sultan M.A., Du R., Smith T.R., Bernstock J.D. (2023). Global, regional, and national economic consequences of stroke.. Stroke.

[3] Mead G.E., Sposato L.A., Sampaio Silva G., Yperzeele L., Wu S., Kutlubaev M., Cheyne J., Wahab K., Urrutia V.C., Sharma V.K., Sylaja P.N., Hill K., Steiner T., Liebeskind D.S., Rabinstein A.A. (2023). A systematic review and synthesis of global stroke guidelines on behalf of the world stroke organization.. Int. J. Stroke.

[4] Dragoș H.M., Stan A., Pintican R., Feier D., Lebovici A., Panaitescu P.Ș., Dina C., Strilciuc S., Muresanu D.F. (2023). MRI radiomics and predictive models in assessing ischemic stroke outcome—A systematic review.. Diagnostics.

[5] Yassi N., Churilov L., Campbell B.C.V., Sharma G., Bammer R., Desmond P.M., Parsons M.W., Albers G.W., Donnan G.A., Davis S.M. (2015). The association between lesion location and functional outcome after ischemic stroke.. Int. J. Stroke.

[6] Sagnier S., Munsch F., Bigourdan A., Debruxelles S., Poli M., Renou P., Olindo S., Rouanet F., Dousset V., Tourdias T., Sibon I. (2019). The influence of stroke location on cognitive and mood impairment. A voxel-based lesion-symptom mapping study.. J. Stroke Cerebrovasc. Dis..

[7] Munsch F., Sagnier S., Asselineau J., Bigourdan A., Guttmann C.R., Debruxelles S., Poli M., Renou P., Perez P., Dousset V., Sibon I., Tourdias T. (2016). Stroke location is an independent predictor of cognitive outcome.. Stroke.

[8] Zhu H., Zuo L., Zhu W., Jing J., Zhang Z., Ding L., Wang F., Cheng J., Wu Z., Wang Y., Liu T., Li Z. (2022). The distinct disrupted plasticity in structural and functional network in mild stroke with basal ganglia region infarcts.. Brain Imaging Behav..

[9] Zuo L., Dong Y., Hu Y., Xiang X., Liu T., Zhou J., Shi J., Wang Y. (2023). Clinical features, brain-structure changes, and cognitive impairment in basal ganglia infarcts: A pilot study.. Neuropsychiatr. Dis. Treat..

[10] Weaver N.A., Kuijf H.J., Aben H.P., Abrigo J., Bae H.J., Barbay M., Best J.G., Bordet R., Chappell F.M., Chen C.P.L.H., Dondaine T., van der Giessen R.S., Godefroy O., Gyanwali B., Hamilton O.K.L., Hilal S., Huenges Wajer I.M.C., Kang Y., Kappelle L.J., Kim B.J., Köhler S., de Kort P.L.M., Koudstaal P.J., Kuchcinski G., Lam B.Y.K., Lee B.C., Lee K.J., Lim J.S., Lopes R., Makin S.D.J., Mendyk A.M., Mok V.C.T., Oh M.S., van Oostenbrugge R.J., Roussel M., Shi L., Staals J., del C Valdés-Hernández M., Venketasubramanian N., Verhey F.R.J., Wardlaw J.M., Werring D.J., Xin X., Yu K.H., van Zandvoort M.J.E., Zhao L., Biesbroek J.M., Biessels G.J. (2021). Strategic infarct locations for post-stroke cognitive impairment: A pooled analysis of individual patient data from 12 acute ischaemic stroke cohorts.. Lancet Neurol..

[11] Langer K.G., Bogousslavsky J. (2020). The merging tracks of anosognosia and neglect.. Eur. Neurol..

[12] Spanò B., Nardo D., Giulietti G., Matano A., Salsano I., Briani C., Vadalà R., Marzi C., De Luca M., Caltagirone C., Santangelo V. (2022). Left egocentric neglect in early subacute right-stroke patients is related to damage of the superior longitudinal fasciculus.. Brain Imaging Behav..

[13] Lafitte R., Jeager M., Piscicelli C., Dai S., Lemaire C., Chrispin A., Davoine P., Dupierrix E., Pérennou D. (2023). Spatial neglect encompasses impaired verticality representation after right hemisphere stroke.. Ann. N. Y. Acad. Sci..

[14] Raimondo L., Oliveira A.F., Heij J., Priovoulos N., Kundu P., Leoni R.F., van der Zwaag W. (2021). Advances in resting state fMRI acquisitions for functional connectomics.. Neuroimage.

[15] Yue X., Li Z., Li Y., Gao J., Han H., Zhang G., Li X., Shen Y., Wei W., Bai Y., Xie J., Luo Z., Zhang X., Wang M. (2023). Altered static and dynamic functional network connectivity in post-stroke cognitive impairment.. Neurosci. Lett..

[16] Li F., Lu L., Shang S., Chen H., Wang P., Muthaiah V.P., Yin X., Chen Y.C. (2021). Altered static and dynamic functional network connectivity in post-traumatic headache.. J. Headache Pain.

[17] Calhoun V.D., Miller R., Pearlson G., Adalı T. (2014). The chronnectome: Time-varying connectivity networks as the next frontier in fMRI data discovery.. Neuron.

[18] Rahaman M.A., Damaraju E., Saha D.K., Plis S.M., Calhoun V.D. (2022). Statelets: Capturing recurrent transient variations in dynamic functional network connectivity.. Hum. Brain Mapp..

[19] Vidaurre D., Llera A., Smith S.M., Woolrich M.W. (2021). Behavioural relevance of spontaneous, transient brain network interactions in fMRI.. Neuroimage.

[20] Liu Y., Zhao X., Tang Q., Li W., Liu G. (2022). Dynamic functional network connectivity associated with musical emotions evoked by different Tempi.. Brain Connect..

[21] Lurie D.J., Kessler D., Bassett D.S., Betzel R.F., Breakspear M., Kheilholz S., Kucyi A., Liégeois R., Lindquist M.A., McIntosh A.R., Poldrack R.A., Shine J.M., Thompson W.H., Bielczyk N.Z., Douw L., Kraft D., Miller R.L., Muthuraman M., Pasquini L., Razi A., Vidaurre D., Xie H., Calhoun V.D. (2020). Questions and controversies in the study of time-varying functional connectivity in resting fMRI.. Netw. Neurosci..

[22] Li Y., Qin B., Chen Q., Chen J. (2022). Altered dynamic functional network connectivity within default mode network of epileptic children with generalized tonic-clonic seizures.. Epilepsy Res..

[23] Espinoza F.A., Liu J., Ciarochi J., Turner J.A., Vergara V.M., Caprihan A., Misiura M., Johnson H.J., Long J.D., Bockholt J.H., Paulsen J.S., Calhoun V.D. (2019). Dynamic functional network connectivity in Huntington’s disease and its associations with motor and cognitive measures.. Hum. Brain Mapp..

[24] Bonkhoff A.K., Espinoza F.A., Gazula H., Vergara V.M., Hensel L., Michely J., Paul T., Rehme A.K., Volz L.J., Fink G.R., Calhoun V.D., Grefkes C. (2020). Acute ischaemic stroke alters the brain’s preference for distinct dynamic connectivity states.. Brain.

[25] van der Horn H.J., Vergara V.M., Espinoza F.A., Calhoun V.D., Mayer A.R., van der Naalt J. (2020). Functional outcome is tied to dynamic brain states after mild to moderate traumatic brain injury.. Hum. Brain Mapp..

[26] Salman M.S., Du Y., Lin D., Fu Z., Fedorov A., Damaraju E., Sui J., Chen J., Mayer A.R., Posse S., Mathalon D.H., Ford J.M., Van Erp T., Calhoun V.D. (2019). Group ICA for identifying biomarkers in schizophrenia: ‘Adaptive’ networks *via* spatially constrained ICA show more sensitivity to group differences than spatio-temporal regression.. Neuroimage Clin..

[27] Wang C., Qin W., Zhang J., Tian T., Li Y., Meng L., Zhang X., Yu C. (2014). Altered functional organization within and between resting-state networks in chronic subcortical infarction.. J. Cereb. Blood Flow Metab..

[28] Wang S., Cai H., Cao Z., Li C., Wu T., Xu F., Qian Y., Chen X., Yu Y. (2021). More than just static: Dynamic functional connectivity changes of the thalamic nuclei to cortex in parkinson’s disease with freezing of gait.. Front. Neurol..

[29] Ma Z.Z., Wu J.J., Hua X.Y., Zheng M.X., Xing X.X., Li S.S., Shan C.L., Ding W., Xu J.G. (2021). Tracking whole-brain connectivity dynamics in the resting-state fMRI with post-facial paralysis synkinesis.. Brain Res. Bull..

[30] Zhou Q., Zhang L., Feng J., Lo C.Y.Z. (2019). Tracking the main states of dynamic functional connectivity in resting state.. Front. Neurosci..

[31] Vicentini J.E., Weiler M., Casseb R.F., Almeida S.R., Valler L., de Campos B.M., Li L.M. (2021). Subacute functional connectivity correlates with cognitive recovery six months after stroke.. Neuroimage Clin..

[32] Cai S., Chong T., Peng Y., Shen W., Li J., von Deneen K.M., Huang L. (2017). Altered functional brain networks in amnestic mild cognitive impairment: A resting-state fMRI study.. Brain Imaging Behav..

[33] Zhao Z., Wu J., Fan M., Yin D., Tang C., Gong J., Xu G., Gao X., Yu Q., Yang H., Sun L., Jia J. (2018). Altered intra- and inter-network functional coupling of resting-state networks associated with motor dysfunction in stroke.. Hum. Brain Mapp..

[34] Zuo L.J., Li Z.X., Zhu R.Y., Chen Y.J., Dong Y., Wang Y.L., Zhao X.Q., Zhang Z.J., Sachdev P., Zhang W., Wang Y.J. (2018). The relationship between cerebral white matter integrity and cognitive function in mild stroke with basal ganglia region infarcts.. Sci. Rep..

[35] Bhatia K.P., Marsden C.D. (1994). The behavioural and motor consequences of focal lesions of the basal ganglia in man.. Brain.

[36] Starkstein S.E., Jorge R.E., Robinson R.G. (2010). The frequency, clinical correlates, and mechanism of anosognosia after stroke.. Can. J. Psychiatry.

[37] Desowska A., Turner D.L. (2019). Dynamics of brain connectivity after stroke.. Rev. Neurosci..

[38] Li Q.G., Zhao C., Shan Y., Yin Y.Y., Rong D.D., Zhang M., Ma Q.F., Lu J. (2020). Dynamic neural network changes revealed by voxel-based functional connectivity strength in left basal ganglia ischemic stroke.. Front. Neurosci..

[39] Li Z., Hu J., Wang Z., You R., Cao D. (2022). Basal ganglia stroke is associated with altered functional connectivity of the left inferior temporal gyrus.. J. Neuroimaging.

[40] Zhang X., Yang Y., Kuai H., Chen J., Huang J., Liang P., Zhong N. (2022). Systematic fusion of multi-source cognitive networks with graph learning - A study on fronto-parietal network.. Front. Neurosci..

[41] Caldinelli C., Cusack R. (2022). The fronto-parietal network is not a flexible hub during naturalistic cognition.. Hum. Brain Mapp..

[42] Braaß H., Gutgesell L., Backhaus W., Higgen F.L., Quandt F., Choe C., Gerloff C., Schulz R. (2023). Early functional connectivity alterations in contralesional motor networks influence outcome after severe stroke: A preliminary analysis.. Sci. Rep..

[43] Rehme A.K., Eickhoff S.B., Rottschy C., Fink G.R., Grefkes C. (2012). Activation likelihood estimation meta-analysis of motor-related neural activity after stroke.. Neuroimage.

[44] Rehme A.K., Fink G.R., von Cramon D.Y., Grefkes C. (2011). The role of the contralesional motor cortex for motor recovery in the early days after stroke assessed with longitudinal FMRI.. Cereb. Cortex.

[45] Bonkhoff A.K., Schirmer M.D., Bretzner M., Etherton M., Donahue K., Tuozzo C., Nardin M., Giese A.K., Wu O., D Calhoun V., Grefkes C., Rost N.S. (2021). Abnormal dynamic functional connectivity is linked to recovery after acute ischemic stroke.. Hum. Brain Mapp..

[46] Lefaucheur J.P., Aleman A., Baeken C., Benninger D.H., Brunelin J., Di Lazzaro V., Filipović S.R., Grefkes C., Hasan A., Hummel F.C., Jääskeläinen S.K., Langguth B., Leocani L., Londero A., Nardone R., Nguyen J.P., Nyffeler T., Oliveira-Maia A.J., Oliviero A., Padberg F., Palm U., Paulus W., Poulet E., Quartarone A., Rachid F., Rektorová I., Rossi S., Sahlsten H., Schecklmann M., Szekely D., Ziemann U. (2020). Evidence-based guidelines on the therapeutic use of repetitive transcranial magnetic stimulation (rTMS): An update (2014–2018).. Clin. Neurophysiol..

[47] Esposito S., Trojsi F., Cirillo G., de Stefano M., Di Nardo F., Siciliano M., Caiazzo G., Ippolito D., Ricciardi D., Buonanno D., Atripaldi D., Pepe R., D’Alvano G., Mangione A., Bonavita S., Santangelo G., Iavarone A., Cirillo M., Esposito F., Sorbi S., Tedeschi G. (2022). Repetitive transcranial magnetic stimulation (rTMS) of dorsolateral prefrontal cortex may influence semantic fluency and functional connectivity in fronto-parietal network in mild cognitive impairment (MCI).. Biomedicines.

